# Risk Identification and Prediction of Coal Workers’ Pneumoconiosis in Kailuan Colliery Group in China: A Historical Cohort Study

**DOI:** 10.1371/journal.pone.0082181

**Published:** 2013-12-23

**Authors:** Fuhai Shen, Juxiang Yuan, Zhiqian Sun, Zhengbing Hua, Tianbang Qin, Sanqiao Yao, Xueyun Fan, Weihong Chen, Hongbo Liu, Jie Chen

**Affiliations:** 1 School of Public Health, China Medical University, Shenyang, Liaoning, P.R. China; 2 School of Public Health, Hebei United University, Tangshan, Hebei, P.R. China; 3 Occupational Disease Prevention and Treatment Hospital of Kailuan Colliery Group, Tangshan, Hebei, P.R. China; 4 School of Public Health, Tongji Medical College, Huazhong University of Science and Technology, Wuhan, Hubei, P.R. China; Universität Bochum, Germany

## Abstract

**Background:**

Prior to 1970, coal mining technology and prevention measures in China were poor. Mechanized coal mining equipment and advanced protection measures were continuously installed in the mines after 1970. All these improvements may have resulted in a change in the incidence of coal workers’ pneumoconiosis (CWP). Therefore, it is important to identify the characteristics of CWP today and trends for the incidence of CWP in the future.

**Methodology/Principal Findings:**

A total of 17,023 coal workers from the Kailuan Colliery Group were studied. A life-table method was used to calculate the cumulative incidence rate of CWP and predict the number of new CWP patients in the future. The probability of developing CWP was estimated by a multilayer perceptron artificial neural network for each coal worker without CWP. The results showed that the cumulative incidence rates of CWP for tunneling, mining, combining, and helping workers were 31.8%, 27.5%, 24.2%, and 2.6%, respectively, during the same observation period of 40 years. It was estimated that there would be 844 new CWP cases among 16,185 coal workers without CWP within their life expectancy. There would be 273.1, 273.1, 227.6, and 69.9 new CWP patients in the next <10, 10-, 20-, and 30- years respectively in the study cohort within their life expectancy. It was identified that coal workers whose risk probabilities were over 0.2 were at high risk for CWP, and whose risk probabilities were under 0.1 were at low risk.

**Conclusion/Significance:**

The present and future incidence trends of CWP remain high among coal workers. We suggest that coal workers at high risk of CWP undergo a physical examination for pneumoconiosis every year, and the coal workers at low risk of CWP be examined every 5 years.

## Introduction

Coal workers who work in underground mines are known to develop a number of diseases, including arthritis, pruritus, chronic bronchitis, chronic obstructive pulmonary disease, and coal workers’ pneumoconiosis (CWP) [1]–[3]. CWP is the most common occupational disease in coal workers [4]–[6]. It is also a serious occupational disease worldwide, especially in developing countries [7]–[11]. When coal workers develop CWP, they not only lose their livelihood, but also have a shorter life expectancy. Pneumoconiosis is also related to certain malignant diseases, such as tuberculosis, mesothelioma, etc [12]–[13]. CWP not only affects patients and their families, but also affects society [14]–[16]. So it is critical to prevent coal workers exposed to dust from developing CWP [17].

In earlier years, coal mining technology and prevention measures in China were poor. A large number of underground coal workers were exposed to high concentrations of dust, and the incidence pneumoconiosis was high. Mechanized coal mining equipment was gradually installed in the mines, and dust level eventually decreased after 1970. These improvements may have resulted in a change in the incidence of CWP. Therefore, it is important to determine the incidence of CWP under these new existing conditions in order to more effectively prevent and control CWP.

Previous researches have shown that there is a downward trend in the incidence of CWP in both developing and developed countries [4], [18]–[21]. Findings indicate that pneumoconiosis can be effectively prevented by some methods, although the disease is not curable at present. Remarkable benefits on the prevention and control of CWP can be achieved in developing countries if positive and effective measures are taken. In developed countries, reducing the number of coal workers exposed to dust has played a crucial role in addition to effective dust control measures [22]–[24]. However, in China, coal accounts for 60% of the primary energy source, and there are about 6 million underground coal workers [25]–[26]. The number of coal workers cannot be reduced. These workers are all at risk of pneumoconiosis, although at low dust exposure levels. The main focus of CWP prevention is to prevent coal workers from developing CWP, with early identification and diagnosis of the disease [27]. Because of this need, it is important to identify the characteristics of CWP at present, and the trends in the incidence of CWP in the near future. Moreover, a feasible and effective prediction model is also needed to predict the risk for CWP in order to take appropriate precautionary measures [28]–[29]. Different preventative and health care schemes could be developed for coal workers at different risks for CWP. Reducing high risk exposure levels for coal workers at high risk for CWP and lengthening the interval between physical examinations for coal workers at low risk for CWP would reduce the financial burden for the health care system and for the government [30].

The Kailuan Colliery Group has a 100-year history of coal mining. Its coal mining processes have included manual work, blast-mining, and general mining. After 1970, a machine mining process, wet operation, and mechanical ventilation were adopted. After 1980, an automated machinery mining process was added. Dust prevention measures in the workplace were also further strengthened after 1990 [31]. This company’s experience represents a typical coal mine in China. A historical cohort study was conducted in the Kailuan Colliery Group. Our main purpose was to explore the incidence of CWP since 1970 as well as the future incidence trends for CWP and identify the risk level for CWP among the coal workers without the disease. This will help to provide scientific evidence and reasonable decisions for further prevention and control of CWP.

## Materials and Methods

### Study Population

The historical cohort study included 16,185 coal workers and 838 patients with CWP who were registered in employment records in the Kailuan Colliery Group, which included personnel files, individual medical records, and occupational records between January 1, 1970 and December 31, 2011. We collected historical data on work history and newly diagnosed pneumoconiosis until December 31, 2011. The study was approved by the Medical Ethics Committee of China Medical University (permit number CMU2009-TA-04). Because written consent from patients was not found to be necessary, it was specifically waived by the Medical Ethics Committee.

We established 4 subcohorts by occupational category (tunneling, mining, combining, and helping). Coal workers, including retired workers, undergo examination for pneumoconiosis every 2 to 3 years by the Kailuan Colliery Group. Coal workers were included in our study as long as they started dust exposure from January 1, 1970 to December 31, 2010 with the Kailuan Colliery Group and had been exposed to dust for at least 1 year after 1970. Those coal workers who left employment were also included if they had been exposed to dust in the mine for 1 or more years. All enrolled workers needed to have physical examination cards and detailed records of occupational history as well as posterior-anterior chest radiographs.

The database included demographic details, work history record with the date of dust exposure, individual medical and pneumoconiosis diagnosis records, and the dust concentrations of the workplace. For the coal workers, observed years started on the first day of dust exposure and ended on the date when the worker was failed to follow-up, or the study ended (December 31, 2011). For the CWP patients, observed years started on the first day of dust exposure and ended on the date when they were diagnosed with CWP, which equals to the latency period. The date of diagnosis of CWP was included in the database. The demographic details and work history records were obtained from personnel files in the human resource section of the Kailuan Colliery Group. The individual medical and pneumoconiosis diagnosis records were obtained from the Occupational Disease Prevention and Treatment Hospital of the Kailuan Colliery Group. The data on dust concentrations in the workplace were obtained from the Department of Dust Detection and Monitoring of the Kailuan Colliery Group.

### Diagnosis of Pneumoconiosis

The diagnosis of CWP was based on the “Diagnostic criteria of pneumoconiosis” and corresponding standard films of pneumoconiosis of China [32]. Chest radiographs of subjects were read and the diagnosis was made independently by 5 qualified experts who were members of the pneumoconiosis diagnosis committee. Diagnosis judgment principle was that the minority should be subordinate to the majority, if the diagnosis results were different by the 5 experts.

### Occupational Category

Four types of working areas in the underground mine in the Kailuan Colliery Group were defined: tunneling, mining, combining, and helping. The tunneling area job included pneumatic drilling, blasting, or lashing of the hard rock materials to create tunnels. The mining area job included drilling, blasting, cutting, and loading coal in the coal area. The combining area was area where the proportion of rock accounted for 20% to 80% in coal layers for excavation. The helping area referred to the area of maintenance, transportation, and electromechanical equipment, where dust was not produced directly. The occupational category was determined by reviewing the work history of the subjects [29], [33]. The workers who engaged consistently in the same working area were defined as tunneling, mining, combining, or helping by their working area titles, respectively. Duration of dust exposure for a coal worker was the sum of years of each dust exposure job. Duration of each dust exposure job was calculated by taking the time from the starting date to the ending date. The workers who had experienced several jobs were classified as tunneling if their tunneling duration was more than half of the whole duration of dust exposure. They would be classified as mining workers if they were involved in tunneling less than 2 years and their mining duration was more than half of the whole duration of dust exposure. The combining workers were those whose duration of tunneling was more than 2 years but not more than half of the whole duration of dust exposure. The helping workers were those who could not be included in any of the tunneling, mining, or combining categories.

### Dust Exposure Data

Dust detection and monitoring were the routine work of the Department of Dust Detection and Monitoring of Kailuan Colliery Group. Dust sampling was done randomly in the tunneling, mining, combining, and helping areas. Dust was sampled twice monthly for each sampling area. Dust concentration and free silica content were measured by gravimetric method and pyrophosphate method, which are the national standard methods [34]–[36]. These numerical data were collected to calculate the geometric means of each area yearly, which were then used to calculate the cumulative dust exposure for each coal worker.

Cumulative dust exposure (CDE) was available for every coal worker [37]. It is a semi-quantitative measure of the cumulative exposure to dust, which is the duration in years multiplied by the dust concentration at the same time in every period of dust exposure for each subject. It includes the total jobs of dust exposure held by the individual during his work history. CDE is given in milligrams/cubic meter-years.

### Statistical Analysis

The cumulative incidence rate was calculated by life table method in the corresponding observed years for different cohorts [18], [28]. The rates were analyzed by Peto log rank test. We analyzed the cumulative incidence rate for the coal workers in different occupational categories (tunneling, mining, combining, and helping), different durations of dust exposure (<20 and 20- years), and different cumulative dust exposure (<100, 100-, and 1000- mg-years).

To predict the possible number of new cases of CWP in the future, the annual average incidence rate, the age distribution of coal workers without CWP, and the life expectancy of local inhabitants were used. To calculate the annual average incidence rate of CWP, dust exposed miners were divided into 8 subgroups according to years of first dust exposure and occupational categories. The life-table method was used to calculate cumulative incidence rate for dust exposed workers of each subgroup, and the ratio of the cumulative incidence rate and follow-up time was the annual average incidence rate of CWP. Using every 5 years as an age group, the number of coal workers exposed to dust were calculated in each subgroup, respectively. Assuming that the average annual incidence rate of each subgroup in the future was at the present level, and the life expectancy of coal workers exposed to dust was equal to the life expectancy of the local male residents, the number of new CWP cases could be predicted.

A three-layer neural network model of multilayer perceptron artificial neural network (MLP-ANN) was constructed to predict the risk for CWP of coal workers without CWP [38]–[43]. The database of CWP patients and workers without CWP were taken as the modeling dataset. The data were divided into 2 subsets on a ratio of 7∶3 at random: training set and validation set. The MLP-ANN contained 4 input variables (occupational category, years of first dust exposure, duration of dust exposure, and cumulative dust exposure) and 1 output neuron (0, absence of CWP; 1, presence of CWP). The importance of each input variable was used to evaluate the contribution of each input variable to predict the risk for CWP. Every coal worker without CWP would obtain a risk probability for developing pneumoconiosis. The occupational characteristics and the probability values predicted were used to categorize coal workers for their level of risk for CWP. The model of MLP-ANN was built by IBM SPSS Modeler 14.1 (SPSS Institute, Inc., Chicago, IL, USA).

## Results

### Baseline Characteristics

A total of 17,023 coal workers were included in the study. Among them, there were 838 patients with CWP, and 16,185 coal workers. For the 838 CWP workers, their average age of onset of CWP was 52.0±4.3, their average duration of dust exposure was 24.8±7.1 years, their latency period was 29.1±5.3 years, and their average age of first dust exposure was 23.0±5.0. For 16,185 coal workers without CWP, their average age in 2011 was 45.6±8.6, their average duration of dust exposed was 23.0±10.4 years, their average observed years was 26.8±9.4 years, and their average age of first dust exposure was 21.4±4.0. The dust concentration decreased with time in different work areas (Table 1). Free silica contents of dust were 22.3%±11.8%, 8.1%±4.5%, 14.7%±8.6% in the tunneling, mining, and combining areas respectively. There were statistically significant differences between the dust exposed workers with CWP and without CWP for occupational category, years of first dust exposure, duration of dust exposure, and cumulative dust exposure as analyzed by the chi square test (Table 2).

**Table 1:** The geometric mean (+1 SD, −1 SD) of dust concentration in the working areas in different years (mg/m^3^).

Year	Tunnelingarea	Miningarea	Combiningarea	Helpingarea
1970-	72.6 (218.4,24.2)	82.1 (200.9,33.5)	51.1 (106.3,24.6)	0.8 (1.1,0.6)
1980-	47.2 (89.9,24.8)	64.0 (126.1,32.5)	47.6 (66.3,34.2)	0.7 (0.8,0.6)
1990-	44.4 (94.4,20.8)	36.0 (65.4,19.8)	26.8 (42.9,16.8)	0.4 (0.5,0.3)
2000-	23.5 (40.2,13.8)	23.9 (29.4,19.4)	15.8 (35.7,7.0)	0.2 (0.5,0.2)

**Table 2:** Characteristics of coal workers with and without coal workers’ pneumoconiosis (CWP).

Characteristics		With CWP(n = 838) (%)	Without CWP(n = 16185) (%)	?^2^	P
Occupational category	Tunneling	248 (29.6)	1137 (7.0)	1168.1	<0.001
	Mining	245 (29.2)	2559 (15.8)		
	Combining	259 (30.9)	2022 (12.5)		
	Helping	86 (10.3)	10467 (64.7)		
Years of first dust exposure	1970-	710 (84.7)	5854 (36.2)	806.0	<0.001
	1980-	112 (13.4)	5798 (35.8)		
	1990-	16 (1.9)	4533 (28.0)		
Duration of dust exposure in years	<10	27 (3.2)	2485 (15.4)	200.2	<0.001
	10-	146 (17.4)	3051 (18.9)		
	20-	435 (51.9)	5031 (31.1)		
	30-	230 (27.4)	5618 (34.7)		
Cumulative dust exposure in mg-year	<100	88 (10.5)	10513 (65.0)	1383.0	<0.001
	100-	238 (28.4)	3237 (20.0)		
	1000-	512 (61.1)	2435 (15.0)		

### Cumulative Incidence Rate of CWP

**Figure 1:**
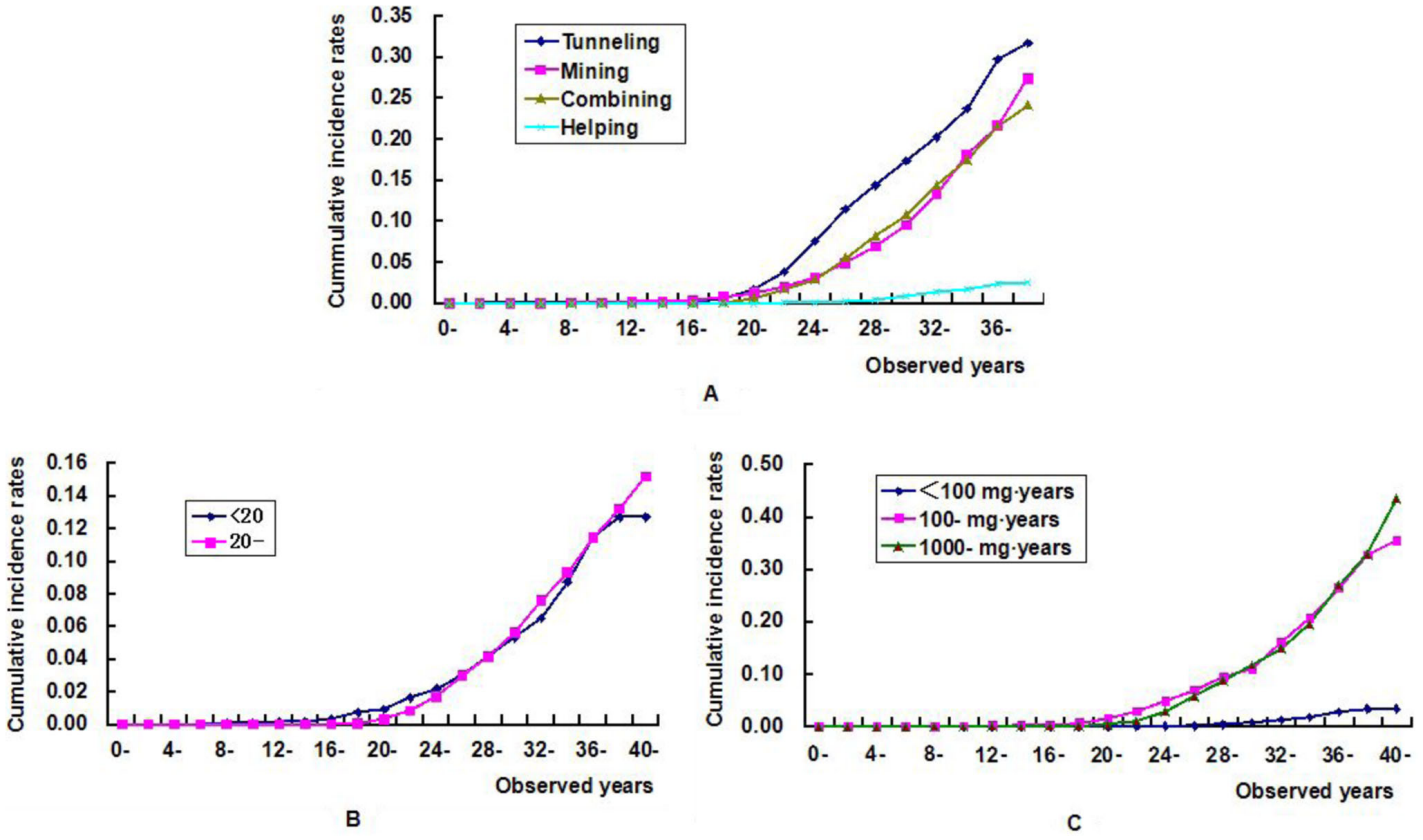
Cumulative incidence rate of coal workers’ pneumoconiosis (CWP) among coal workers. (A) Cumulative incidence rate of CWP in different occupational categories: tunneling cohort vs mining cohort (χ^2^ = 26.3, P<0.001), tunneling cohort vs combining cohort (χ^2^ = 6.5, P = 0.011), tunneling cohort vs helping cohort (χ^2^ = 942.9, P<0.001), mining cohort vs combining cohort (χ^2^ = 7.3, P = 0.007), mining cohort vs helping cohort (χ^2^ = 565.5, P<0.001), and combining cohort vs helping cohort (χ^2^ = 797.6, P<0.001); (B) Cumulative incidence rate of CWP in different durations of dust exposure: <20 years group vs 20- years group (χ^2^ = 0.1, P = 0.716); (C) Cumulative incidence rate of CWP in different cumulative dust exposure: <100 mg-years group vs 100- mg-years group (χ^2^ = 637.7, P<0.001), <100 mg-years group vs 1000- mg-years group (χ^2^ = 767.3, P<0.001), and 100- mg-years group vs 1000- mg-years group (χ^2^ = 0.3, P = 0.576).

The cumulative incidence rates of CWP for tunneling, mining, combining, and helping workers were 31.8%, 27.5%, 24.2%, and 2.6%, respectively, during the same observation period of 40 years (Figure 1A). The cumulative incidence rate of the tunneling worker cohort was higher than those of the mining worker cohort (χ^2^ = 26.3, P<0.001), the combining worker cohort (χ^2^ = 6.5, P = 0.011), and the helping worker cohort (χ^2^ = 942.9, P<0.001). The cumulative incidence rate of the mining worker cohort was higher than that of the helping worker cohort (χ^2^ = 565.5, P<0.001). The cumulative incidence rate of the combining worker cohort was higher than those of the mining worker cohort (χ^2^ = 7.3, P = 0.007) and the helping worker cohort (χ^2^ = 797.6, P<0.001). Among the coal workers with different durations of dust exposure (<20 and 20- years), the cumulative incidence rates of CWP were 12.7% and 15.2%, respectively, during the same observation period of 42 years (Figure 1B). The cumulative incidence rates were 43.5%, 35.5%, and 3.5% among the coal workers whose cumulative dust exposures were 1000- mg-years, 100- mg-years, and <100 mg-years, respectively, during the same observation period of 42 years (Figure 1C). The cumulative incidence rate of the <100 mg-years group was lower than those of the 100- mg-years group (χ^2^ = 637.7, P<0.001), and the 1000- mg-years group (χ^2^ = 767.2, P<0.001).

### Incidence Trend of New CWP in the Future

The average annual incidence rates of CWP among the 1970- group were 8.1‰ (tunneling), 8.0‰ (mining), 6.5‰ (combining), and 0.6‰ (helping), respectively. And among the 1980- group, the rates were 1.7‰ (tunneling), 2.1‰ (mining), 1.4‰ (combining), and 0.3‰ (helping), respectively.

The average annual incidence rate and life expectancy of the coal workers were used to calculate the possible number of new CWP cases in the future. Based on this, there would be about 844 new CWP patients among 16,185 coal workers without CWP. The predicted numbers of new CWP patients were 174, 317, 213, and 139 among tunneling, mining, combining, and helping workers, respectively. New CWP patients were mainly concentrated in the age of 40 to 60 group, with a proportion of about 87.0% (734 of 844) (Table 3). There would be 273.1, 273.1, 227.6, and 69.9 new CWP patients in the next <10, 10-, 20-, and 30- years respectively in the study cohort within their life expectancy (Table 4).

**Table 3:** Predicted number of new coal workers’ pneumoconiosis (CWP) cases at different ages among coal workers without CWP within their life expectancy.

Occupational category	n	Predicted number of new CWP at different ages (y)						Total
		<20 (5)	20–29 (1162)	30–39 (2338)	40–49 (6099)	50–59 (6497)	60–(84)	(16185)
Tunneling	1137	0	2.2	6.8	37.4	127.7	0.3	174.4
Mining	2559	0	16.2	29.1	100.9	169.8	1.0	317.0
Combining	2022	0.2	2.8	11.6	47.5	148.7	2.4	213.1
Helping	10467	0.1	14.6	22.5	39.5	61.9	0.6	139.2
Total (%)	16185	0.2 (4.0)	35.7 (3.1)	69.8 (2.9)	225.1 (3.7)	508.5 (7.8)	4.4 (5.2)	843.6 (5.2)

**Table 4:** Predicted number of new coal workers’ pneumoconiosis (CWP) cases in future years among coal workers without CWP within their life expectancy.

Occupational category	n	Predicted number of new CWP in future years(yrs)				Total
		<10	10–19	20–29	30-	
Tunneling	1137	58.5	58.5	50.0	7.5	174.4
Mining	2559	100.2	100.2	85.1	31.4	317.0
Combining	2022	72.6	72.6	56.4	11.4	213.1
Helping	10467	41.7	41.7	36.1	19.6	139.2
Total	16185	273.1	273.1	227.6	69.9	843.6

### Identification and Classification of Risk Levels for CWP

We built the prediction model of MLP-ANN and repeated to train the model until the accuracy of model was stable. The variables of input layer were the occupational category, years of first dust exposure, duration of dust exposure, and cumulative dust exposure. The importance of input variables was shown by the fact that cumulative dust exposure accounted for 0.39, occupational category accounted for 0.35, years of first dust exposure accounted for 0.25, and duration of dust exposure accounted for 0.01 (Figure S1). The hidden layer of the final model included 74 synapses. The accuracy of the model achieved was 91.8% (15627 of 17023) at the cut-off of 0.2. The sensitivity of the model achieved was 81.4% (682 of 838). The specificity of the model achieved was 92.3% (14945 of 16185).

Based on the prediction of new 844 cases of CWP in the future years, we considered to increase the protected number by 50% (844+844×50% = 1266) for expanding the scope of protection of high risk for CWP. In order to fit about 1266 of the scope of protection, we defined 0.2- as the high risk probability for CWP. Because the cumulative incidence rate of CWP in the 1980- group was 2.8%, and has declined obviously. The cumulative incidence rate of CWP for helping workers was 2.6%, much lower than those of tunneling, mining, and combining workers (31.8%, 27.5%, 24.2%, respectively). There were 6869 coal workers started to expose to dust after 1980 in helping cohort. We considered that they were at low risk for CWP. In order to fit about 6869 of the low risk level, we defined <0.1 as the low risk probability for CWP. As shown in Table 5, we divided coal workers without CWP into 3 groups according to the probability values of their outputs, as follows, <0.1 (6255 subjects), 0.1- (8690 subjects), and 0.2- (1240 subjects). Subjects whose risk probabilities were over 0.2 were at high risk for CWP, whose risk probabilities were between 0.1 and 0.2 were at middle risk for CWP, and whose risk probabilities were under 0.1 were at low risk for CWP. The rates of high risk for CWP were 17.9%, 15.9%, 20.0%, and 2.1%, and the rates of low risk were 4.1%, 13.6%, 16.8%, and 52.7%, in the tunneling, mining, combining, and helping groups of occupational category, respectively. The rates of high risk for CWP were 16.9%, 4.2%, and 0.1%, and the rates of low risk were 12.5%, 31.2%, and 82.4%, in the 1970-, 1980-, and 1990- groups of years of first dust exposure, respectively. The rates of high risk for CWP were 4.2%, 8.3%, 6.7%, and 10.1%, and the rates of low risk were 65.0%, 68.1%, 26.6%, and 19.18%, in <10, 10-, 20-, and 30- years groups of duration of dust exposure, respectively. The rates of high risk for CWP were 2.1%, 14.2%, and 22.8%, and the rates of low risk were 52.8%, 16.7%, and 6.9%, in <100 mg-years, 100- mg-years, and 1000- mg-years groups of cumulative dust exposure, respectively.

**Table 5:** Predicted number (%) at different risk probability of coal workers’ pneumoconiosis (CWP) in the future for coal workers without CWP.

Characteristics		n	Predicted number (%) at different risk probability		
			<0.1 (6255)	0.1- (8690)	0.2- (1240)
Occupational category	Tunneling	1137	47(4.1)	886(77.9)	204(17.9)
	Mining	2559	348(13.6)	1805(70.5)	406(15.9)
	Combining	2022	340(16.8)	1277(63.2)	405(20.0)
	Helping	10467	5520(52.7)	4722(45.1)	225(2.1)
Years of first dust exposure	1970-	5875	732(12.5)	4151(70.7)	992(16.9)
	1980-	5798	1807(31.2)	3747(64.6)	244(4.2)
	1990-	4512	3716(82.4)	792(17.6)	4(0.1)
Duration of dust exposure (yrs)	<10	2739	1780(65.0)	843(30.8)	116(4.2)
	10-	3101	2112(68.1)	733(23.6)	256(8.3)
	20-	5153	1372(26.6)	3437(66.7)	344(6.7)
	30-	5192	991(19.1)	3677(70.8)	524(10.1)
Cumulative dust exposure (mg-year)	<100	10513	5547(52.8)	4741(45.1)	225(2.1)
	100-	3237	539(16.7)	2238(69.1)	460(14.2)
	1000-	2435	169(6.9)	1711(70.3)	555(22.8)

## Discussion

The incidence of pneumoconiosis has been a decreasing trend, but it is still a most serious occupational disease worldwide. Pneumoconiosis prevention remains a top priority in developing countries [4], [7], [10], [44]. Although dust concentrations have decreased gradually in coal mines [37], [45], dust has not been completely controlled. The incidence rates of CWP have declined obviously for recent decades. However, the cumulative incidence rates of CWP for tunneling, mining, and combining workers were 31.8%, 27.5%, and 24.2%, respectively, and much higher than that of helping workers. This result was similar with other literature [28]. Our results showed that dust concentrations in Kailuan Colliery Group have also decreased obviously for recent decades, but the dust concentrations of tunneling, mining, combining areas were still much higher than the occupational exposure limits (OELs) [46]. In China, the descending tendency of dust concentrations was similar among coal mines. Dust concentrations in Kailuan Colliery Group were lower than those of some coal mines (such as Lingquan Colliery Group and Datong Colliery Group), and higher than those of some other coal mines (such as Huainan Colliery Group and Laiwu Colliery Group). Therefore, it is necessary to take further actions to control dust [47].

We predicted that about 844 new cases among 16,185 coal workers without CWP were likely to develop CWP within their life expectancy. Based on the prediction of new cases of CWP in the future years, we predicted the future risk probability for CWP of every coal worker without CWP by a three-layer neural network. We identified the different risk levels for CWP by the variables of the years of occupational category, first dust exposure, duration of dust exposure, and cumulative dust exposure. Coal workers whose risk probabilities were over 0.2 were classified as high risk level for CWP, whose risk probabilities were between 0.1 and 0.2 were classified as middle risk level for CWP, and whose risk probabilities under 0.1 were classified as low risk level for CWP. The classification of low risk level in our study was a relative designation. We thought that although the risks of CWP for some coal workers were lower than others by occupational category or duration of dust exposure, the low risks were much higher than the general working population. We predicted that the rates of high risk level for CWP of the tunneling, mining, and combining groups were much higher (15.9–20.0%) than that of helping group (2.1%) by occupational categories. The rate of high risk level for CWP of 1970- group was much higher (16.9%) than those of 1980- and 1990- groups (4.2%, 0.1%) by years of first dust exposure. The rate of low risk level for CWP of helping group was much higher (52.7%) than those of tunneling, mining, and combining groups (4.1–16.8%) by occupational categories. The rate of low risk level for CWP of 1990- group was much higher (82.4%) than those of 1970- and 1980- groups (12.5%, 31.2%) by years of first dust exposure. The rates of low risk level for CWP of <10 and 10- year groups were much higher (65.0% and 68.1%) than those of 20- and 30- year groups (26.6% and 19.1%) by duration of dust exposure. We could shorten the interval of regular examinations for the coal workers at high risk for CWP, and prolong the interval of regular examinations for the low risk coal workers. The risk probability prediction for CWP could provide scientific basis for the secondary prevention (early identification, diagnosis, and treatment of the disease) [27], [48].

The “Guideline of occupational health surveillance (GBZ188-2007)” regulates that the silica-exposure workers should be examined once a year if silica dust level is over the national health standard. The silica-exposure workers should be examined every two years if silica dust level is below the national health standard. The coal workers should be examined every two years if coal dust level is over the national health standard. The coal workers should be examined every three years if coal dust level is below the national health standard. The ex-coal workers should be examined every five years [49].

The coal workers have been examined for pneumoconiosis every 2 to 3 years in the Kailuan Colliery Group. The “Guideline of occupational health surveillance (GBZ188-2007)” of China regulates that the dust exposed workers should be examined for pneumoconiosis every 1 to 5 years according to the dust level, free silica content, and the duration of dust exposure [49]. The coal workers at high risk for CWP were mainly the tunneling, mining, combining workers, who started to expose to dust before 1980. Those workers should undergo a physical examination every year. The coal workers at low risk for CWP were mainly the helping workers, who started to expose to dust after 1990, and whose duration of dust exposure was less than 20 years. Those workers should undergo a physical examination every 5 years. Therefore, we suggest that coal workers at high risk for CWP should undergo a physical examination every year, coal workers at low risk for CWP be examined every 5 years, and coal workers at middle risk for CWP may still be examined every 2 to 3 years. The risk probability prediction provides the scientific basis for amending the regulation of occupational health surveillance [50].

## Supporting Information

Figure S1 The importance of input variables in the prediction course of multilayer perceptron artificial neural network.
(TIF)
