# Supplementary figures and images for: Risk Identification and Prediction of Coal Workers’ Pneumoconiosis in Kailuan Colliery Group in China: A Historical Cohort Study
Source: PLoS One. 2013 Dec 23;8(12):e82181. doi: 10.1371/journal.pone.0082181 (PMC3871577; doi:10.1371/journal.pone.0082181)

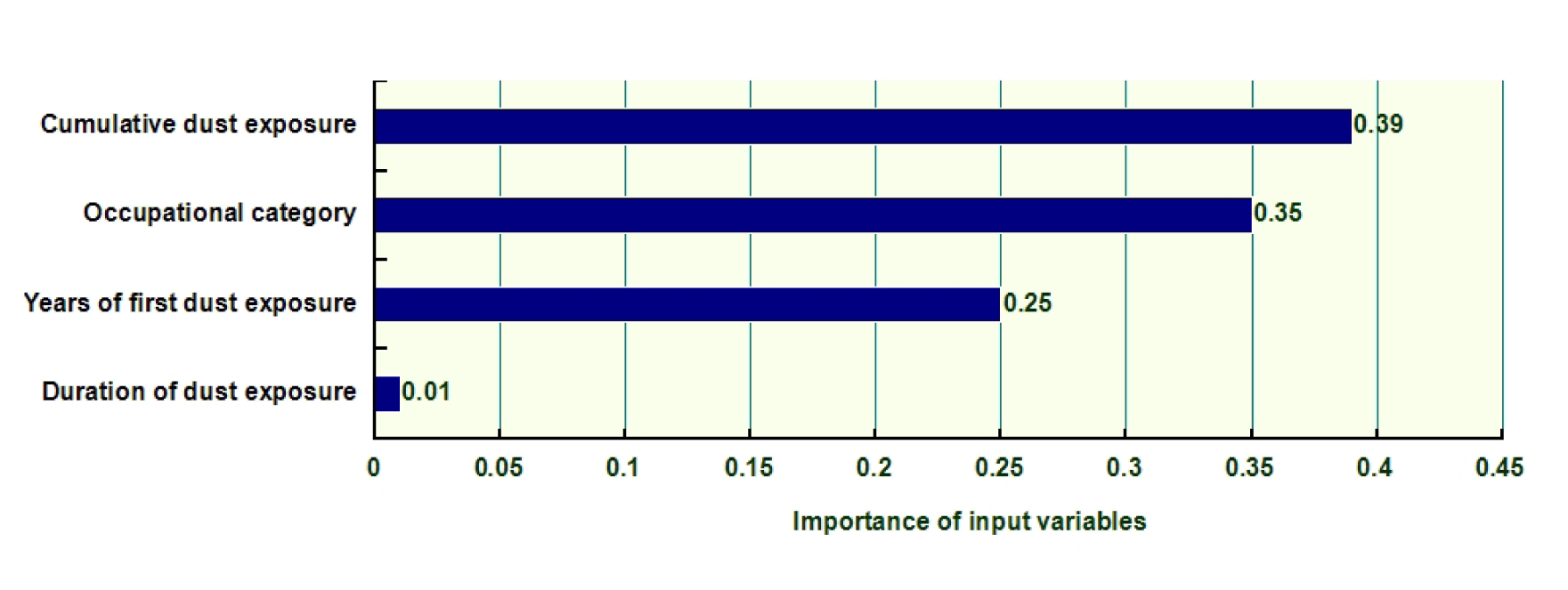

Supplement: Figure S1 — The importance of input variables in the prediction course of multilayer perceptron artificial neural network. (TIF) [file pone.0082181.s001.tif]
